# Supplementary material for: The phased pan-genome of tetraploid European potato
Source: Nature. 2025 Apr 16;642(8067):389–97. doi: 10.1038/s41586-025-08843-0 (PMC12158759; doi:10.1038/s41586-025-08843-0)
Supplement: Supplementary file 5 — Supplementary Figs. 3–14, 20, 21, 23–27 and 34–44. [file 41586_2025_8843_MOESM5_ESM.zip › suppl_figure_21/suppl_figure_21d_chr04.pdf]

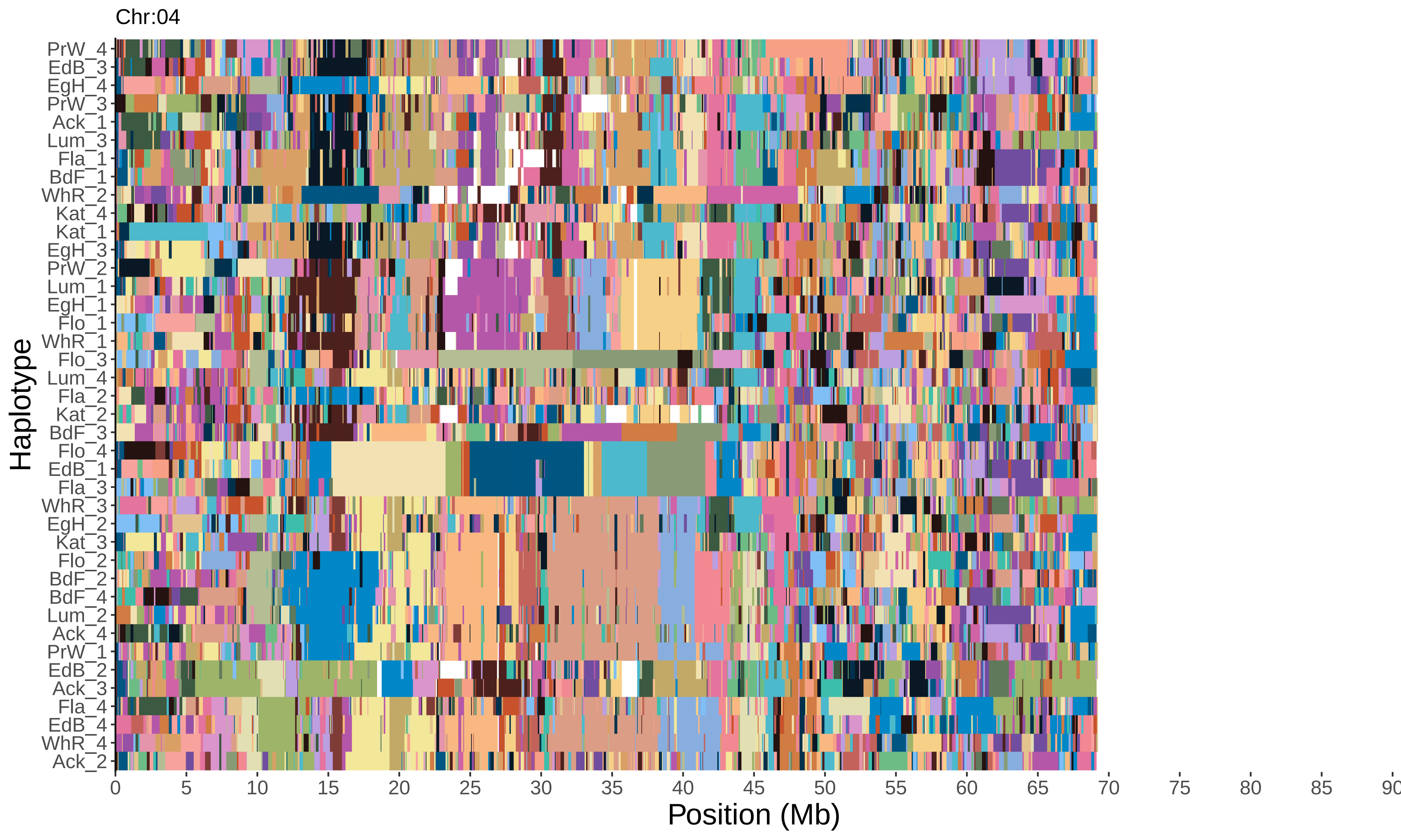

Chr:04
Haplotype
PrW_4
EdB_3
EgH_4
PrW_3
Ack_1
Lum_3
Fla_1
BdF_1
WhR_2
Kat_4
Kat_1
EgH_3
PrW_2
Lum_1
EgH_1
Flo_1
WhR_1
Flo_3
Lum_4
Fla_2
Kat_2
BdF_3
Flo_4
EdB_1
Fla_3
WhR_3
EgH_2
Kat_3
Flo_2
BdF_2
BdF_4
Lum_2
Ack_4
PrW_1
EdB_2
Ack_3
Fla_4
EdB_4
WhR_4
Ack_2
0
5
10
15
20
25
30
35
40
45
50
55
60
65
70
75
80
85
90
Position (Mb)
